# A Simulation and Training Platform for Remote-Sighted Assistance

**DOI:** 10.3390/s24237773

**Published:** 2024-12-04

**Authors:** Xuantuo Huang, Rong Zhang, Yancheng Li, Bingao Zhang, Jianhua Zhang, Jingjing Xu, Shengyong Xu

**Affiliations:** 1 Key Laboratory for the Physics and Chemistry of Nanodevices, School of Electronics, Peking University, Beijing 100871, China; xuantuo@pku.edu.cn (X.H.); a2000012737@stu.pku.edu.cn (R.Z.); lycheng@pku.edu.cn (Y.L.); zhangbingao@pku.edu.cn (B.Z.); 2 School of Computer Science and Engineering, Tianjin University of Technology, Tianjin 300384, China; 3 School of Integrated Circuits, Shandong University, Jinan 250100, China

**Keywords:** assistive technology, human–computer interaction, remote-sighted assistance, simulation and training, visually impaired people

## Abstract

Remote-sighted assistance (RSA) is a technology designed to provide assistance for visually impaired people (VIPs). In this scene, a remote-sighted agent communicates and sends commands to navigate and assist VIPs via real-time video sent back. However, the latency in real-time video and the deviation in the execution of instructions by VIPs are two important factors that affect the performance of agents to guide them. Therefore, how to enable agents to better guide VIPs under conditions of video transmission latency and deviation in instruction execution is an important issue. In this paper, we utilize Unreal Engine to create a virtual training platform for RSA, which simulates VIPs executing instructions in the real world and resembles the environment in RSA systems. We aim to help remote-sighted agents quickly master the set of vibration commands formed after encoding tactile vibrations and enable them to guide VIPs more effectively. Our experiment results show that, compared with untrained novices, when guiding people through the same path, agents trained on this platform reduce their average time by 32.09% and their average number of contacts with the environment by 57.57%. Our work provides agents with a simple and convenient simulation and training platform designed to enhance their performance by guiding VIPs with less travel time and fewer environmental contacts. Through this platform, agents can more effectively assist the visually impaired.

## 1. Introduction

Globally, there are nearly 340 million people with moderate or above visual impairment, of whom, 43.3 million are completely blind [1]. Hence, it is important to develop visual restoration and assistive technologies to improve the quality of life for people with visual impairments. In recent years, remote-sighted assistance (RSA) has emerged as a new technology to offer support for people with visual impairments [2]. The RSA system can be roughly divided into two parts: visually impaired people (VIPs), i.e., users, and remote-sighted assistants, i.e., agents [3]. A user captures the real-time video of the surroundings via a smartphone [4], a wearable digital camera [5,6], or a web camera [5], and then transmits it to an agent. Based on the visual information offered by the video, the agent provides guidance and assistance to the user according to the user’s request.

Conventional electronic travel aids (ETAs) focus on solving the difficulties of VIPs in avoiding obstacles, wayfinding, and key object recognition [7]. While ETAs are incredibly effective in certain scenarios, they also have severe limitations in complex environments where contextual or detailed visual information is necessary [8]. For example, they might not be able to interpret visual signs, recognize faces, provide detailed descriptions of objects, or implement these functions at the same time. Traditional ETAs offer VIPs a significant level of independence; however, they are only partially effective in navigating for VIPs and helping them gain a comprehensive understanding of their environment. This is probably because vision loss uniquely affects individuals, presenting challenges distinct from other disabilities due to humans’ primary reliance on visual perception, [9,10], navigation [11,12], daily living skills [13,14], communication [15], social interactions [16,17], etc. Therefore, most ETAs are unable to fully meet the desires and requirements of VIPs [5], and none of these assistive technologies have become widespread [18]. In the RSA system, the remote agents, being human, can avoid many of the shortcomings of traditional ETAs while also being able to offer better assistance tailored to the user’s needs, as the RSA system can be seen as a combination of human intelligence and traditional ETAs [2]. Many related prototypes or industrial products have been proposed in recent years, such as VizWiz [19], BeSpecular [20], BeMyEyes [21], Aria [22], TapTapSee [23], Crowdviz [24], etc.

In existing RSA-related research, agents assist users by sending instructions; the format of these instructions is one of the key issues [3]. Existing work mainly utilizes texts [25], synthetic speech [26], natural communication [4,21,22], and vibrotactile feedback [27,28]. Compared to other forms of instructions, vibratory tactile feedback has a unique advantage since humans naturally have a sense of orientation to touch perception and can respond to it quickly [29,30]. Moreover, tactile feedback is not affected by environmental disturbances, and it can still be used even in a noisy or crowded environment, and it does not disturb others. Moreover, although the human tactile system perceives relatively less information compared to other senses [31], the tactile sensitivity of human skin can be encoded to increase the capacity for information, thereby conveying more information [32,33].

However, both users and agents are initially unfamiliar with the RSA system and the tactile-encoded instructions. After receiving tactile feedback, users often execute the instructions inaccurately. And delays in transmitting live video and the user’s response make it difficult for agents to properly guide users using tactile feedback. Therefore, both users and agents need to undergo training to gradually master it [34]. In this way, users can learn how to use the RSA system, and agents can learn how to guide users more effectively. But learning in a real environment can be risky and troublesome. One solution to this issue is to create a virtual training platform, allowing agents and users to train in a safe, risk-free, virtual, yet realistic space. Existing works on virtual training for VIPs are limited, with only a few studies leveraging virtual reality (VR) technology within Unity to support VIPs in using traditional ETAs [35,36]. These efforts provide a safe environment for VIPs to familiarize themselves with ETAs, but they focus solely on traditional ETAs and do not address the unique characteristics of RSA systems, such as video transmission latency, and inaccuracies in instruction execution. To the best of our knowledge, no virtual training systems specifically designed for RSA have been developed. Motivated by this gap, we propose a simulation platform tailored to RSA systems. Our system is designed to improve the performance of agents rather than VIPs through training. The purpose of this work is to reduce the impact of the latency of video transmission and inaccuracy in instruction execution on the agent so that the agent can better assist the visually impaired.

Our contributions are as follows: First, we identified that system latency in the RSA and deviations in the execution of commands sent by agents to VIPs are two crucial factors affecting the agents’ ability to guide them. Therefore, we utilized the inertial measurement unit (IMU) to collect data on how VIPs respond to tactile feedback commands. Additionally, we employed Unreal Engine to offer a virtual training environment for agents within the RSA system and reappear the execution of commands by VIPs in this platform. By implementing system latency and instruction execution deviations in Unreal Engine, this platform enables agents to realistically grasp the behaviors of real users. Moreover, we implemented a method to mark the user’s real-time position in the system, and through experiments, we demonstrated that marking the user’s real-time position in the live video stream could potentially reduce the impact of system latency on the agent. Finally, we validated our system’s ability to enhance the agent’s performance in guiding VIPs through on-site experiments. The results indicate that our research indeed offers a simulated training platform for RSA, thus better enabling agents to provide navigation and assistance to VIPs.

## 2. Methods

### 2.1. System Overview

**Figure 1:**
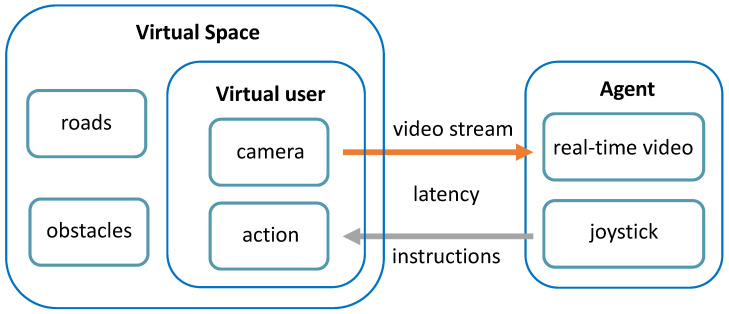
Virtual training platform architecture. We created a virtual space where roads and obstacles can be randomly generated. A virtual user in the RSA system walks into the virtual space and avoids obstacles. The agent can see the real-time video sent back by the camera on the virtual user and send instructions through the joystick to guide the virtual user. In this process, we used latency to simulate the time consumption of wireless transmission in the actual RSA system.

As illustrated in Figure 1, we developed an experimental simulation and training platform using Unreal Engine for RSA, which provides a preliminary framework for evaluating, developing, and refining. By leveraging such a platform, we can simulate the functionality of agents observing real-time video transmitted from the user’s camera and guiding the user with tactile feedback instructions. Additionally, it can also simulate the corresponding actions taken by users upon receiving tactile feedback.

In this work, we designed a scenario in which a user, represented as 180 Unreal units tall (equivalent to 180 cm in the real world), wore a helmet equipped with a camera mounted on their head. The agent could see the viewpoint captured by the camera, simulating the real-time video transmitted from the camera worn by the user, mirroring what the agent would see in reality. Here, we chose the head as the viewpoint solely to maintain consistency with the experiments discussed later. In fact, in this platform, we could set the viewpoint at any position and freely adjust the camera’s field of view, thus simulating any RSA scenario. Based on the real-time video transmission delay (about 0.9 s, as the delay of real-time video is usually less than 1 s), the system’s transmission delay was set to 1.5 s (including 0.6s of average reaction time, as shown in the experiments below). We utilized gait resources within Unreal Engine to add a walking gait for the user, aiming to closely replicate the scene an agent would see through the video as realistically as possible.

### 2.2. Scene Construction

**Figure 2:**
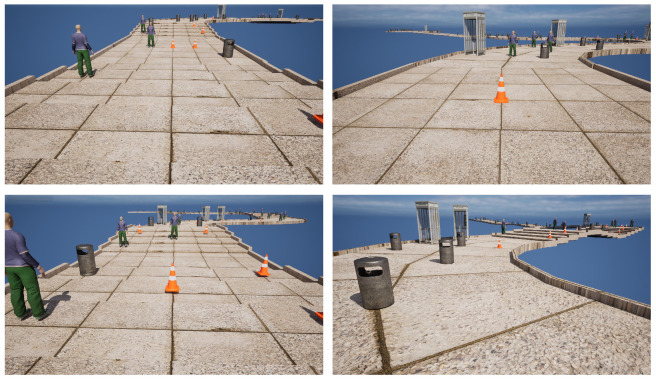
A simulation and training platform for RSA. Using Unreal Engine, a simulated pedestrian street scene was constructed, featuring paths for going straight, turning left, turning right, and ascending and descending steps. The scene includes four types of obstacles: trash cans, telephone booths, road barriers, and pedestrians.

**Figure 3:**
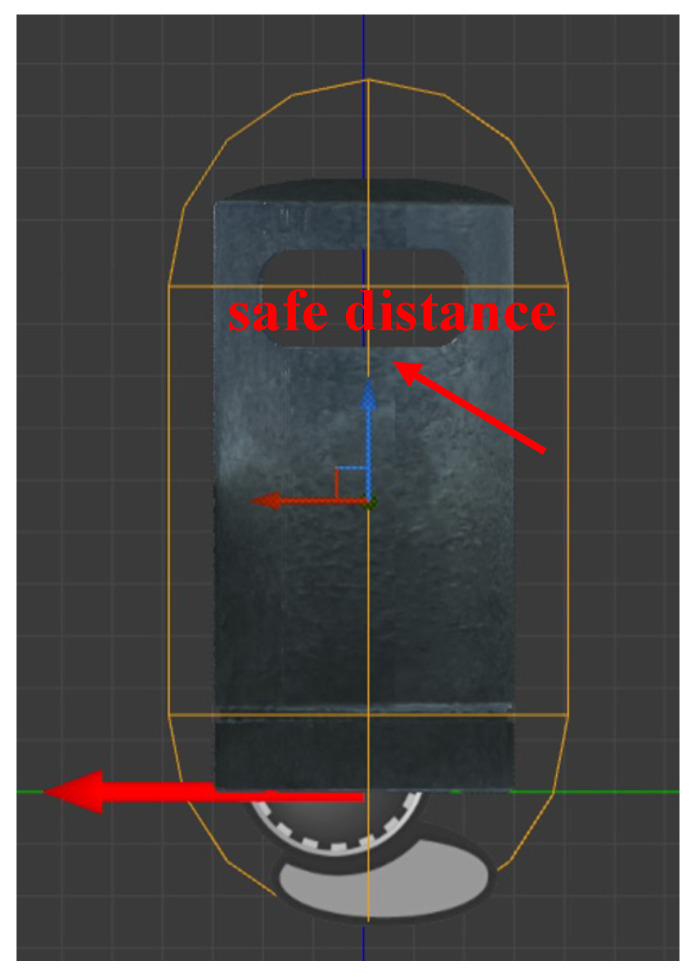
We propose the concept of a safety distance for obstacles to train agents to guide users in a softer manner.

In our work, we chose to simulate scenarios in which visually impaired individuals walked on streets, randomly generating straight paths, left-turn roads, right-turn roads, and stairs going up and down, as shown in Figure 2. Simultaneously, obstacles were generated on these roads, including pedestrians, roadblocks, trash cans, and telephone booths—four types of obstacles that are common on living streets. The user in the system moved along the street and responded to received instructions with corresponding actions. Meanwhile, the agent had to guide the user forward and avoid obstacles by using the given set of tactile-encoded instructions, ensuring no contact with obstacles. Additionally, we incorporated the concept of a safety distance into this process, as illustrated in Figure 3. During the process of avoiding obstacles, the agent should ensure that the user maintains a safe distance from the obstacles, maintaining a safe distance. This is because we want the agent to guide the user in a softer manner, not just to avoid collisions between the user and obstacles. In our system, the safety distance is set to 10 Unreal units (equivalent to 10 cm in the real world). If the distance between the user and an obstacle is less than 10 cm, it is considered that the user has come into contact with the obstacle. By establishing a safety distance, we aim to help the agent guide VIPs in a more standardized and effective manner.

### 2.3. Calibration of Real Position

**Figure 4:**
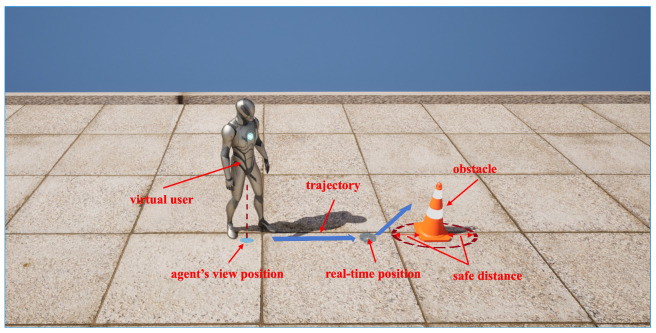
Due to the transmission time of the live video and the time it takes for the user to process instructions, the user will continue to move forward after the agent issues a command. The user will not execute the instruction to avoid obstacles until they reach their real-time position.

In the RSA system, a sighted agent assists visually impaired individuals by viewing the real-time video transmitted back to them. In this architecture, the time it takes for network transmission results in a discrepancy between the real-time video images viewed by the sighted agent and the real-time location of the visually impaired person. When the visually impaired person is walking, the image seen by the sighted agent is actually lagging behind the user’s real-time position, with a lag distance of $s=v*t_{delay}$, where *v* denotes the user’s walking speed and $t_{delay}$ denotes the network transmission delay plus the time it takes for the user to process instructions. This means that agents need to make judgments and send instructions in advance when guiding visually impaired individuals. If it is possible to mark the user’s real-time position in the video seen by the agent, this could help the agent guide the user more effectively. However, it is often difficult to achieve this in practice, so we hope that this aspect can be reflected in the process of training agents, allowing agents to learn to make judgments in advance when guiding visually impaired people. As illustrated in Figure 4, the real-time location of the visually impaired person is marked in the system with a blue halo. This halo is always located directly in front of the user, at a distance equal to the user’s walking speed multiplied by the system delay. We also validate that accurately marking the real position benefits the agent in guiding the user. This is demonstrated through experiments where the agent directs users to walk in the center of a virtual straight path environment and actually follow along the track.

### 2.4. RSA System

**Figure 5:**
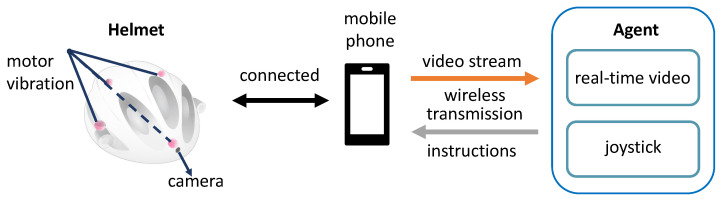
The RSA system architecture we designed involves the visually impaired individual wearing a helmet connected to a mobile phone. The mobile phone wirelessly transmits live video from the helmet’s camera to a remote agent. The agent, in turn, sends directional instructions by manipulating a joystick, which activates motors to produce vibrations in the helmet, guiding the visually impaired person in navigating their surroundings.

To validate our virtual training platform, we designed an RSA system, as illustrated in Figure 5. The visually impaired person (VIP) wears a helmet equipped with a camera and four vibration motors, positioned evenly at the front, back, left, and right. The camera’s video stream is wirelessly transmitted to a remote agent via the mobile phone. After observing the real-time video and making decisions, the agent sends directional commands through a joystick, causing the corresponding motor to vibrate and guiding the VIP’s movement.

We implement the tactile instructions through motor vibrations, as detailed in Table 1. Different combinations of motor vibrations on the helmet correspond to specific instructions, each with a unique meaning. The remote agent uses a joystick to intuitively guide the VIPs by sending these tactile cues. In the next section, we will discuss in detail how these tactile instructions are integrated into the virtual training system.

### 2.5. Specific Implementation of Instructions

In this work, the encoded vibrational feedback instructions and their specific meanings are shown in Table 1, including turn left, turn right, turn left a bit, turn right a bit, move forward, slow down, and stop. These instructions can already meet the basic needs of assisting visually impaired individuals in navigation and obstacle avoidance. Many other related works have tactile instructions similar to or even simpler than those in Table 1 [27,28,37]. In fact, the platform can be configured with a richer and more complex set of tactile-encoded instructions. However, in practical applications, numerous and overly complex feedback of tactile instructions can increase the cognitive load for both users and agents and, therefore, should be considered carefully. Moreover, when visually impaired individuals travel, their walking speed is significantly slower than that of sighted individuals and does not vary greatly. Furthermore, measurements from related research indicate that the range of their speed changes is also quite limited, typically varying between 0.4 to 1 m/s when utilizing ETAs [38,39,40]. This variation is not distinctly noticeable in Unreal Engine. Therefore, in our platform, when the user moves forward, they will progress at a speed of 1 m/s. Upon receiving a slowdown instruction, the speed will reduce to 0.5 m/s, with the acceleration of speed change set to 1 m/s^2^, consistent with normal human walking. This setup derives from the natural condition, where the acceleration in the lumbar and thoracic regions during a regular walk oscillates between 0 and 0.2 g [41]. When visually impaired individuals receive turn instructions (such as turn left, turn right, turn left a bit, turn right a bit), their turning angles do not always align with the preset desired values but instead exhibit some deviations. This is because it is difficult for them to determine their orientation in space due to the lack of reference objects. We wanted our work to reflect this aspect, so we conducted experiments to measure this deviation and integrated the results into our platform. In other words, each time the user in the system receives an instruction to turn, there will be a deviation, and their turning angles will not be precisely the same as the preset instructions. The turning angle is defined as the difference of the user’s yaw angle before and after completing an instruction.

**Figure 6:**
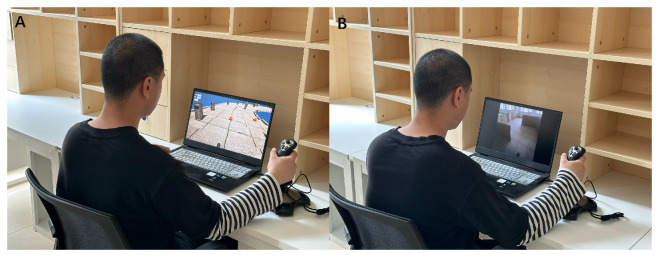
Work scenarios of remote-sighted agents. (**A**) A remote-sighted agent undergoing training on our platform. (**B**) A remote-sighted agent offering assistance for VIPs through our RSA system.

As shown in Figure 6, for the tool used to send instructions, we chose a joystick (LaiShiDa, PXN-2113PRO, PXN Electronic Technology Co., Shenzhen, China) as the tool for agents to send tactile-encoded instructions to users in our work. The advantage of using a joystick as a tool for sending instructions is that it is an intuitive approach. This approach aligns with natural human interactions, leveraging the familiarity and ergonomic design of joysticks to facilitate efficient and precise instruction transmission, thus reducing the cognitive burden on agents. We can track how far the joystick deviates from the center in both horizontal and vertical directions; moreover, the joystick is equipped with multiple buttons that can be pressed. To map the joystick’s operations to commands, we designed a simple state machine. A state transition occurs when the joystick moves to another area identifiable by coordinates, or when a button is pressed. Specific state transitions trigger corresponding commands. In addition, the design of the states is simplified because complex states would require the user to remember specific transition conditions to avoid incorrect operations. Certainly, using a joystick can be applied in a broader range of scenarios, as currently, only a small portion of its capabilities is utilized, and many functions remain undeveloped. In the future, we could use the joystick to implement richer encodings, such as determining emergency situations by reading the acceleration of the joystick when the agent uses it.

### 2.6. Experiments Setup

To evaluate our work, we conducted a series of experiments to demonstrate the effectiveness of the simulation and training platform. First, to study the inaccuracy of VIPs in responding to tactile instructions, we designed a turning experiment (Section 3.1). In this experiment, we measured the deviation in responses from five blindfolded participants (four males and one female) to tactile instructions, including turning left, turning right, turning slightly left, and turning slightly right. This deviation was integrated into our simulation platform as a realistic representation of actual behavior. Next, to validate the hypothesis that annotating the real-time position of VIPs in RSA system video streams can enhance agent performance, we conducted a track scenario test (Section 3.2). In this test, an agent guided four users (two males and two females) along a 1.2-m-wide, 20-m-long track. Since marking the user’s position in real-time video streams is challenging, no real-time position annotations were provided in this scenario. To compare, we also simulated the same track within our platform and allowed the same agent to guide the user along the simulated track under the condition that the real-time position is annotated. The hypothesis was tested by analyzing the proportion of the user’s deviation from the track in both scenarios, validating whether the annotated position improved user guidance accuracy. Finally, to demonstrate the effectiveness of the training platform, we organized an on-site user guidance test (Section 3.3). In this experiment, 12 sighted volunteers (8 males and 4 females) participated as agents to assist a blindfolded user. None of them served as agents before. The agents guided the blindfolded user along a predefined 15-m path using tactile instructions. This path included boundaries, obstacles, and turns, as illustrated in Section 3.3. Each agent guided the user through the path three times under each experimental condition.

## 3. Results

To accurately reflect the response of visually impaired individuals to commands, we first conducted experiments to measure their performance after receiving vibrational feedback and then incorporated the results into the system. Moreover, to demonstrate how marking the user’s real-time position can potentially improve the agent’s performance in guiding users, we conducted a track scenario test. In this experiment, we have the agent guide the user along the track and compare the proportion of times the user exceeds the track boundaries throughout the trial with and without the real-time position being marked. This is because walking straight without veering off course is important in daily life and is difficult for visually impaired individuals to achieve on their own. Finally, to evaluate the effectiveness of the platform in training agents, we also organized on-site trials to compare the performance of agents trained on the platform with those not trained on the platform in guiding users.

### 3.1. Turning Experiment

**Figure 7:**
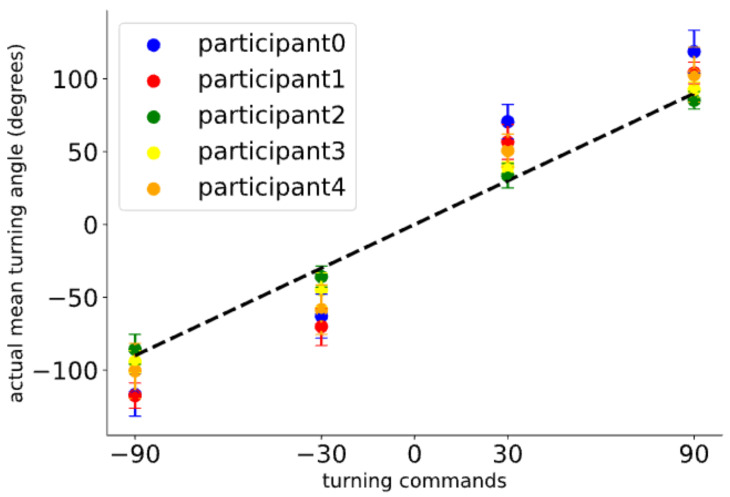
The average turning angle for each participant after receiving a turn instruction, with counterclockwise rotation denoted as positive and clockwise rotation as negative. The error bars represent a deviation of one standard deviation from the mean, and the dashed line indicates the scenario where the expected turning angle matches the actual turning angle.

**Figure 8:**
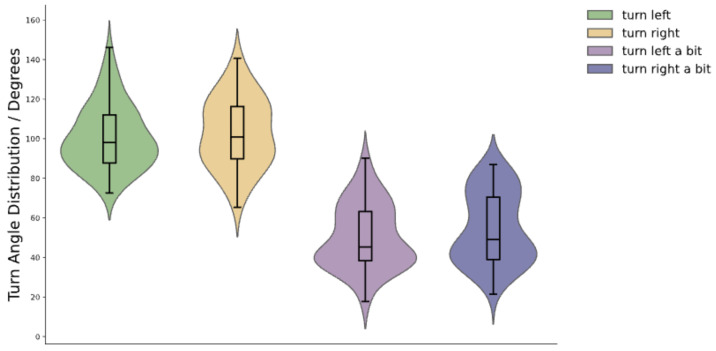
The distribution for each turn-related instruction. It shows that participants’ actual turning angles are generally larger than the preset values. Each violin plot, with the area enclosed by black edges representing the corresponding box plot, provides detailed information on the median with the thick black band inside each box. The bottom and top of the box identify the first and third quartiles, respectively. The colored area of the violin plots corresponds to the probability density of the data.

Due to the lack of vision, visually impaired individuals lack reference points when receiving turn instructions, which means they cannot accurately complete the turn angles set by the instructions and tend to deviate from the preset value. This makes it challenging for agents to guide users appropriately during navigation. Therefore, we hope our platform can also help agents address this issue. To realistically reflect the inaccuracy with which visually impaired individuals execute tactile feedback instructions, we organized experiments to measure their performance in executing turn instructions. We tested the actual turning angles of five blindfolded participants (four males and one female) after receiving instructions. In the experiment, we tested the instructions related to turning listed in Table 1 (i.e., turn left, turn right, turn left a bit, turn right a bit). Before the test, we informed the participants about the meanings of our instructions. During the test, the participants executed the corresponding actions based on the tactile feedback received. We used an inertial measurement unit (IMU, model IM948 used in the experiment) to measure the participants’ turning angles. We tested each instruction 15 times, totaling 60 trials, with all instructions randomized and distributed in no particular order. All experiments were successfully completed, and no participant’s data were excluded. The results of each participant for a single tactile feedback instruction are depicted in Figure 7. The final average turning angles and standard deviations for all participants for each tactile feedback are shown in Figure 8. We discovered that the turning angles of visually impaired individuals tend to be larger than the preset values after receiving tactile turn instructions. In the system, to simulate the inaccuracy of users executing tactile feedback instructions, we randomly selected a value with equal probability within the range of one standard deviation above and below the average value of each instruction to represent the user’s turning angle.

**Figure 9:**
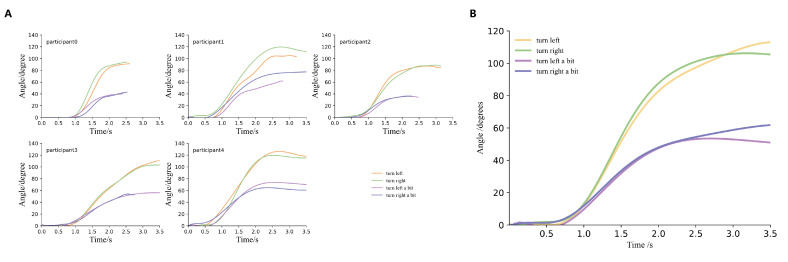
Execution results of tactile feedback instructions. (**A**) The turning angle of each participant changes over time, with the average value calculated for all data points for each instruction per participant. (**B**) The average change in turning angle over time for participants, with the average taken across all participants for each type of instruction. We can see that the turning angles of visually impaired individuals tend to be larger than the preset values after receiving the tactile turn instructions.

As shown in Figure 9, we also captured how the users’ turning angles changed over time after receiving tactile feedback from the instructions. Figure 9A shows the results of each user’s execution of the instructions listed in Table 1, while Figure 9B represents the average execution of instructions from Table 1 by all users. The results indicate that after receiving an instruction, users require some time to react, with an average reaction time of around 0.6 s. Moreover, we can find that users take similar amounts of time to complete ‘turn left’ and ‘turn right’ instructions, and similarly, ‘turn left a bit’ and ‘turn right a bit’ take comparable but shorter times than full turns. It is also observed that the angles of turning left and turning right are similar, as are the angles for turning right a bit and turning left a bit. This indicates that users show no significant difference in results when turning the same angle to the left or right.

### 3.2. Track Scenario Test

**Figure 10:**
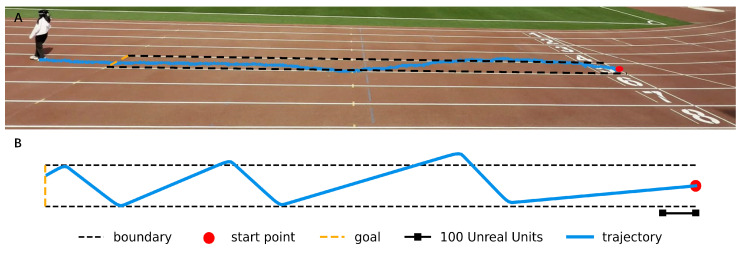
Track scenario test. (**A**) In the absence of visual cues indicating the real-time position of the user, an agent guides the user to walk along a track that is 1.2 m wide and 20 m long. The YOLOv8 network is used to obtain visualized trajectory maps. (**B**) In Unreal Engine, the real-time position of the user is marked, allowing the agent to guide the user along a track scaled the same as the one in scenario A. From this setup, the visualized trajectory map is shown. In scenario A, the average distance that all subjects went beyond the track boundary accounted for 20.62% of the total distance. In scenario B, the average distance that users went beyond the track boundary accounted for 7.98% of the total distance. This indicates that marking the user’s real position in the video stream can potentially improve the performance of agents in guiding users.

In the RSA scenario, the sighted agent assists visually impaired individuals by watching a real-time video stream. The delay in transmitting real-time video is inevitable, so the scene observed by the agent always lags behind the user’s view. We believe that marking the real-time position of the user in the video would help agents guide the user, however, it is difficult for us to mark the user’s position in the real-time video stream. Therefore, to demonstrate this assumption, we conducted a tracking experiment to assess how well an agent can guide a user walking along a track, focusing on the accuracy of guidance with and without real-time visual cues indicating the user’s position. This includes comparing the proportion of trials deviating from the track in virtual environments (like those created with Unreal Engine) versus real-world settings. The reason for choosing this scenario is that walking straight without veering off course is important in daily life and is difficult for visually impaired individuals to achieve on their own. In both scenarios, the track’s central area is standardized to a width of 1.2 m and a length of 20 m to ensure consistency. In practice, without marking the real-time position of the user, an agent guided four users (two males and two females) to walk on the track. We used the YOLOv8 network [42] to obtain the users’ walking paths, as shown in Figure 10A. Each experiment was repeated four times. Ultimately, in the Unreal Engine simulation, the user deviated from the central area only 7.98% of the time, as shown in Figure 10B, whereas in the real-world track, the user deviated from the central area an average of 20.62% of the time. This indicates that if we could mark the real-time position of the user in the real-world video stream, it would likely reduce the impact of system latency on the agent’s performance to guide the user, potentially leading to better guidance by the agent.

### 3.3. On-Site User Guidance Test

**Figure 11:**
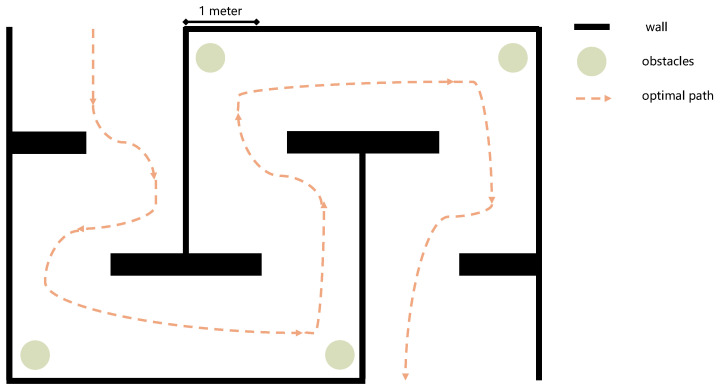
The experimental site’s floor plan, a path with a width of 2.5 m and a total length of 15 m. The yellow dashed line indicates an ideal path that does not come into contact with any obstacles or walls.

As the goal is to provide a training platform for agents within the RSA system, we also conducted on-site tests to demonstrate the training effectiveness of this platform for agents. In this experiment, 12 sighted volunteers (8 males and 4 females) served as agents to assist the same blindfolded user. These agents guided the blindfolded user through the same path using tactile instructions. This path owned boundaries, obstacles, and turns, with a total length of 15 m, as illustrated in Figure 11. Each agent guided the user through the path three times under each experimental condition.

**Figure 12:**
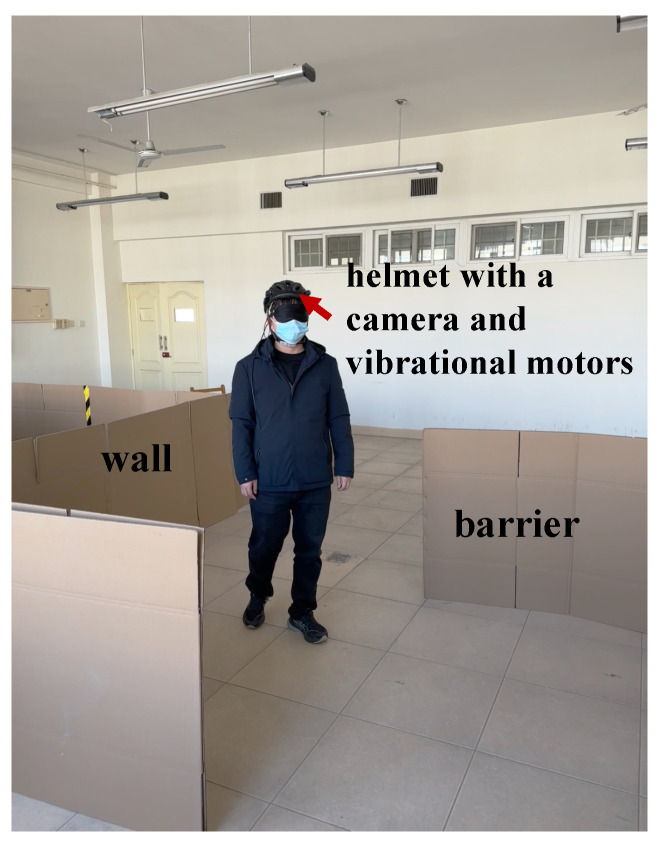
During the actual experimental process, a blindfolded user wearing a helmet equipped with a camera and vibration motors. The camera captures real-time video that is transmitted to the agent via a smartphone. The encoded vibrations from the helmet’s motors provide tactile feedback to the user.

In the experiment, the blindfolded user wore a helmet equipped with a camera and vibration motors, as shown in Figure 12. The camera remotely transmitted the footage to the agent via a smartphone. The four vibration motors, uniformly distributed around the inside of the helmet, formed the instructions listed in Table 1 through the timing and sequence of their vibrations. The blindfolded user was familiar with the instructions and would not execute them incorrectly. These 12 agents had no prior experience with similar training. To better evaluate the effect of the platform on the agents’ performance and improvement, we evenly divided the 12 agents into three groups. Members of Group 1 did not undergo training on the platform and directly served as agents to help the user navigate the path. Members of Group 3 served as agents to assist the user in navigating the path after completing training on the platform. To highlight the importance of accounting for inaccuracies in users’ execution of tactile feedback, we introduced an additional group, referred to as Group 2. After removing the execution errors from the instructions, members of Group 2 served as agents to guide the user through the path (referred to as Group 2 trained without deviation). Subsequently, these members were trained under normal platform conditions. After completing the training, they guided the user through the path (referred to as Group 2 trained with deviation).

During training, the system randomly generates obstacles on the path at regular intervals. The agent needs to guide the user in the system to move forward and avoid these obstacles. The system records the time taken for the user to complete the path and the number of times the user comes into contact with the environment. During training, the system randomly generates obstacles on the path at regular intervals. The agent needs to guide the user in the system to move forward and avoid these obstacles. Each time the user progresses a certain distance (equal to the distance at which obstacles are generated), the progress counter increments by one. During this process, if the user’s distance to an obstacle is less than the safety distance (10 cm here), it is considered contact with the obstacle, and this contact is recorded. When the progress reaches 100 and the number of contacts is less than 5 (with accuracy reaching 95%), it is considered that the agent has passed the training and can guide users in the real world through the path shown in Figure 11. If the number of contacts exceeds or equals 5 before the progress reaches 100, then the training will be considered a failure, and the agent will need to undergo retraining until he/she passes.

**Figure 13:**
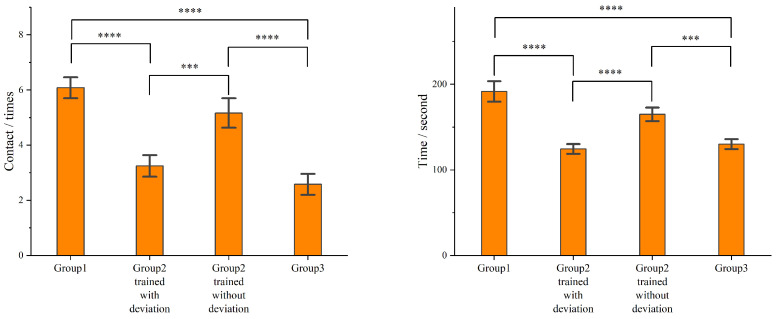
The statistics for the average time and average number of contacts when agents guided users through the path, as shown in Figure 11A, demonstrate that agents who underwent direct training achieved a significant reduction in both the time taken to complete the process and the number of user–environment contacts, compared to untrained agents (reducing by 32.09% and 57.57%, respectively). This indicates that our platform indeed has a good training effect (*** *p* < 5× 10^−3^, **** *p* < 5× 10^−4^).

After the agent passes the training, he/she will guide the blindfolded user through the path in Figure 11 three times. We recorded the time it takes for each agent to guide the user through this path, as well as the number of contacts between the user and the environment. Eventually, we obtained the average time spent and the number of contacts for each group, as shown in Figure 13. The results show that the more contacts the user has with the environment, the longer it often takes for the user to complete the path. This reflects a point that the poorer the performance of the agent in guiding the user.

Based on our analysis of the results, compared to untrained novices (Group 1), agents trained through this training platform (Group 3) have reduced their average time by 32.09% and decreased the number of contacts by 57.57%. This indicates that our platform is highly effective in training agents. Compared to Group 1, Group 2 trained without deviation did not show a significant improvement in guiding the user in terms of time spent and the number of contacts (time increased by 13.96%, the number of contacts increased by 14.97% (less than one time), with a P value greater than 0.05). This indicates that in such scenarios, deviations in how visually impaired individuals execute tactile commands significantly impact agents’ performance in guiding them. Thus, the RSA must introduce the inaccuracy of user execution of instructions. In other words, common VR games cannot achieve our goal of improving the performance of agents in guiding users. It is necessary to introduce key features of the RSA system into such a system. Meanwhile, Group 2 trained with deviation had similar times and contact numbers in guiding users compared to Group 3. This indicates that the training effect of our platform is independent of the selected agents because the participants acting as agents in these two groups are completely different. We believe that the improvement in the performance of agents mainly comes from their proficiency. Through our developed simulation and training platform, they are not only more familiar with the instructions but also have better expectations of the users’ execution results. They can anticipate what will happen next, thus generally enhancing the agents’ performance in guiding users.

## 4. Discussion

Although there is now a lot of research aimed at helping visually impaired people with mobility, what we commonly see in our daily life is that visually impaired individuals use white canes and guide dogs, with less frequent sightings of them using related assistive mobility products. From our perspective, this is because visually impaired individuals are not familiar with and lack trust in related products. In other words, visually impaired individuals often need to go through a long period of use and training before they might gradually accept a new assistive mobility product. In recent years, there has been related work that utilizes VR technology to allow visually impaired individuals to train with ETAs in virtual spaces [35]. However, compared to traditional ETA devices, which focus on visually impaired individuals as end-users, agents in the RSA system play a unique role by leveraging human intelligence. Therefore, the RSA system has received considerable attention from researchers in recent years. But it is challenging for agents to guide users due to the latency in real-time video transmission and the uncertainty of visually impaired individuals in executing commands. These aspects have often been overlooked in previous research. For this purpose, we constructed a virtual space simulating the RSA system, where agents are trained on our platform to address the issue of agents needing a longer time to understand and familiarize themselves with tactile commands. This approach also avoids potential risks and troubles without the need to involve real visually impaired individuals. At the same time, this platform can be quickly distributed to end-users, providing a reliable basis for large-scale training of agents. Experimental results also indicate that our work can improve the performance of agents in guiding users.

### 4.1. Motion Construction and Instruction

In our current work, the modeling of the user’s movement is now preliminary; for example, the walking speed of visually impaired individuals does not change. In the future, we can consider more complex dynamic modeling to simulate the subtle variations in the walking speed of visually impaired individuals. Moreover, this work only investigated a subset of typical tactile instructions that can assist users in walking on outdoor pedestrian paths. In such a simulated RSA virtual space, we can attempt to simulate additional tactile instructions to represent a wider range of scenarios. Furthermore, audio is another important form of interaction in the RSA system. Our current work mainly focuses on the tactile feedback form of commands. In future work, we could explore adding voice recognition capabilities to the system, converting the agent’s audio into commands to guide visually impaired individuals. This would create a multimodal training platform, providing a more comprehensive simulation for agent training.

### 4.2. Latency Effect

The transmission latency of the RSA system is a key factor affecting agents’ ability to navigate for the visually impaired. This latency primarily arises from network delays in transmitting real-time video and the user’s processing time for instructions. This leads to the fact that the agent’s judgments often lag behind the user’s actual needs. In this work, it was demonstrated that marking the user’s real-time position in a live video stream can potentially reduce the impact of latency on agents. However, to thoroughly address this issue, it is necessary to establish a 3D map of the environment. If we can create a 3D map, we can obtain more real-time information from it, such as the user’s real-time orientation and position, the planned path to the desired destination, the distance to obstacles, etc., which can reduce the system’s latency impact on the agent and allow the agent to obtain more environmental cues, thereby better guiding the user. Some efforts have already been made to explore this topic [43]. This work developed an offline 3D map of indoor environments and demonstrated its effectiveness in moderate-sized scenarios. Such technologies remain in the early stages of application, especially for large-scale and outdoor environments, where additional challenges in data processing and real-time localization make implementation even more complex. Additionally, in this work, the 3D map and real-time video are presented separately to the agent, without integrating the information. In the future, we can further utilize augmented reality (AR) technology to project all real-time information into the live video stream captured by the camera, which can reduce data transmission volume and support real-time transmission of online maps. In addition, the environmental modeling in our work is merely a prototype for functional demonstration, and the scenarios are not diverse. A realistic 3D map could also help us build richer and more realistic maps to better reflect the environment in which the user is situated.

### 4.3. Tactile Instructions

For visually impaired individuals, both voice prompts and tactile cues can be used to perform tasks such as obstacle avoidance and navigation. Due to their impaired vision, visually impaired individuals rely heavily on hearing and touch to carry out daily activities. Therefore, common RSA systems typically employ voice and tactile encoding for guidance. From our perspective, voice should primarily be used to describe the environment, while tactile encoding should mainly be employed to indicate directional actions (such as turn left, turn left a bit, turn right, turn right a bit). In a typical walking scenario, most actions should be guided using tactile instructions, allowing visually impaired individuals to use their hearing to perceive the surrounding environment and respond to unexpected events, such as car horns or pedestrians shouting. Related research also indicates that, compared to other senses, tactile stimuli can be responded to very quickly [29,30]. As a result, tactile encoding used for action instructions can form conditioned reflexes through simple training, making it easy for visually impaired individuals to learn. We translated the rotation of the joystick into tactile encoding, making it easier for agents to master, just like driving a car. The output instructions are equivalent, which avoids misunderstandings that may arise when agents use voice commands due to unclear speech or accents. Moreover, the applications of tactile encoding are extensive and not limited to RSA systems; it is also widely used in areas such as immersive experiences in virtual reality [44] and medical rehabilitation devices [45].

### 4.4. Experimental Limitations

This study has certain limitations due to the use of sighted, blindfolded participants rather than individuals who are blind. Blindfolded participants may exhibit different walking behaviors and spatial awareness, as those who are blind often develop unique strategies for navigation and obstacle avoidance. For example, blind folders usually walk faster than the visually impaired. Additionally, the participants in this study did not use white canes, which are commonly relied upon by visually impaired individuals to gather tactile information about their surroundings. This absence could impact how participants respond to obstacles and navigate. Another limitation relates to the sample size and diversity of participants, which may influence the generalizability of our findings. A more representative and larger sample could provide deeper insights into the system’s effectiveness across different user profiles.

Despite these limitations, they do not detract from the primary aim of this study, which was to demonstrate the feasibility of our simulation and training platform for the RSA system. These findings serve as an important proof of concept, laying the groundwork for future research with a more diverse participant pool and additional navigational tools.

## 5. Conclusions

To improve the performance of agents in the RSA system, we used Unreal Engine to build a prototype system for simulating RSA. We constructed a pedestrian street scene, including straight paths, left and right turns, and stairs. On the road, we can randomly generate four types of obstacles, containing pedestrians, trash cans, roadblocks, and telephone booths. Moreover, by studying the actual execution process of visually impaired individuals in response to tactile feedback, we realistically simulated their execution deviations from tactile feedback. The commands formed by tactile feedback include the following: turn left, turn right, turn left a bit, and turn right a bit. And we integrated the deviations of these commands into our system. Furthermore, this platform offers a simulated training environment. Agents in the RSA system can be trained to learn how to guide users using tactile feedback. Through organizing trials, our experimental results indicate that our work can indeed improve the performance of agents in guiding users. In our experiments, compared to untrained novices, trained agents reduced the time taken to guide users through the same path by 32.09%, and the number of contacts between the user and the environment decreased by 57.57%. In the future, we believe this technology can enhance the performance of agents in RSA systems. And this improvement will enable RSA technology to better assist more visually impaired individuals.

## Figures and Tables

**Table 1:** Details of instructions.

Instruction	Meaning	Vibrotactile
Turn left	Turn left 90 degrees	The left motor vibrates three times
Turn right	Turn right 90 degrees	The right motor vibrates three times
Turn left a bit	Turn left 30 degrees	The left motor vibrates once
Turn right a bit	Turn right 30 degrees	The right motor vibrates once
Move forward	Go straight	The front motor vibrates once
Slow down	Go straight with a smaller speed	The back motor vibrates three times
Stop	Stop	All motors vibrate simultaneously

## Data Availability

The data presented in this study are available on request from the corresponding author Shengyong Xu.

## References

[1] Bourne R., Steinmetz J.D., Flaxman S., Briant P.S., Taylor H.R., Resnikoff S., Casson R.J., Abdoli A., Abu-Gharbieh E., Afshin A. (2021). Trends in prevalence of blindness and distance and near vision impairment over 30 years: An analysis for the Global Burden of Disease Study. Lancet Glob. Health.

[2] Lee S., Reddie M., Tsai C.H., Beck J., Rosson M.B., Carroll J.M. The emerging professional practice of remote sighted assistance for people with visual impairments. Proceedings of the 2020 CHI Conference on Human Factors in Computing Systems.

[3] Lee S., Yu R., Xie J., Billah S.M., Carroll J.M. Opportunities for human-AI collaboration in remote sighted assistance. Proceedings of the 27th International Conference on Intelligent User Interfaces.

[4] Baranski P., Strumillo P. Field trials of a teleassistance system for the visually impaired. Proceedings of the 2015 8th International Conference on Human System Interaction (HSI).

[5] Bujacz M., Baranski P., Moranski M., Strumillo P., Materka A. Remote guidance for the blind—A proposed teleassistance system and navigation trials. Proceedings of the 2008 Conference on Human System Interactions.

[6] Gleason C., Ahmetovic D., Savage S., Toxtli C., Posthuma C., Asakawa C., Kitani K.M., Bigham J.P. (2018). Crowdsourcing the installation and maintenance of indoor localization infrastructure to support blind navigation. Proc. ACM Interact. Mob. Wearable Ubiquitous Technol..

[7] Real S., Araujo A. (2019). Navigation systems for the blind and visually impaired: Past work, challenges, and open problems. Sensors.

[8] Merugu S., Ghinea G. (2022). A review of some assistive tools and their limitations for visually impaired. Helix- Sci. Explor. Peer Rev. Bimon. Int. J..

[9] Brouwer D.M., Sadlo G., Winding K., Hanneman M.I. (2008). Limitations in mobility: Experiences of visually impaired older people. Br. J. Occup. Ther..

[10] Masal K.M., Bhatlawande S., Shingade S.D. (2024). Development of a visual to audio and tactile substitution system for mobility and orientation of visually impaired people: A review. Multimed. Tools Appl..

[11] Dong J., Karmann C. (2024). A review study of space perception and navigation of people with low vision: Is simulated low vision a reliable methodology?. IOP Conf. Ser. Earth Environ. Sci..

[12] Jain G., Teng Y., Cho D.H., Xing Y., Aziz M., Smith B.A. (2023). “I Want to Figure Things Out”: Supporting Exploration in Navigation for People with Visual Impairments. Proc. ACM Hum.-Comput. Interact..

[13] Haymes S.A., Johnston A.W., Heyes A.D. (2002). Relationship between vision impairment and ability to perform activities of daily living. Ophthalmic Physiol. Opt..

[14] Hoeben M., Langelaan M., Klevering J., Keunen J.E., van Rens G.H. (2020). Low vision rehabilitation for better quality of life in visually impaired adults. Cochrane Database Syst. Rev..

[15] Little J.A., Saunders K. (2015). A lack of vision: Evidence for poor communication of visual problems and support needs in education statements/plans for children with SEN. Public Health.

[16] Klauke S., Sondocie C., Fine I. (2023). The impact of low vision on social function: The potential importance of lost visual social cues. J. Optom..

[17] Kempen G.I., Ballemans J., Ranchor A.V., van Rens G.H., Zijlstra G.R. (2012). The impact of low vision on activities of daily living, symptoms of depression, feelings of anxiety and social support in community-living older adults seeking vision rehabilitation services. Qual. Life Res..

[18] Isaksson J., Jansson T., Nilsson J. (2020). Audomni: Super-scale sensory supplementation to increase the mobility of blind and low-vision individuals—A pilot study. IEEE Trans. Neural Syst. Rehabil. Eng..

[19] Bigham J.P., Jayant C., Miller A., White B., Yeh T. VizWiz: LocateIt-enabling blind people to locate objects in their environment. Proceedings of the 2010 IEEE Computer Society Conference on Computer Vision and Pattern Recognition-Workshops.

[20] Holton B. (2016). BeSpecular: A new remote assistant service. Access World Magazine.

[21] Be My Eyes. https://www.bemyeyes.com/.

[22] Aira. https://aira.io/.

[23] TapTapSee. https://taptapseeapp.com/.

[24] Holton B. (2015). Crowdviz: Remote video assistance on your iphone. Afb Accessworld Magazine.

[25] Lasecki W.S., Wesley R., Nichols J., Kulkarni A., Allen J.F., Bigham J.P. Chorus: A crowd-powered conversational assistant. Proceedings of the 26th Annual ACM Symposium on User Interface Software and Technology.

[26] Petrie H., Johnson V., Strothotte T., Raab A., Michel R., Reichert L., Schalt A. (1997). MoBIC: An aid to increase the independent mobility of blind travellers. Br. J. Vis. Impair..

[27] Chaudary B., Paajala I., Keino E., Pulli P. (2017). Tele-guidance based navigation system for the visually impaired and blind persons. Proceedings of the eHealth 360°: International Summit on eHealth.

[28] Scheggi S., Talarico A., Prattichizzo D. A remote guidance system for blind and visually impaired people via vibrotactile haptic feedback. Proceedings of the 22nd Mediterranean Conference on Control and Automation.

[29] Rosenkranz R., Altinsoy M.E. (2020). Mapping the sensory-perceptual space of vibration for user-centered intuitive tactile design. IEEE Trans. Haptics.

[30] Petermeijer S., Doubek F., De Winter J. Driver response times to auditory, visual, and tactile take-over requests: A simulator study with 101 participants. Proceedings of the 2017 IEEE international conference on systems, man, and cybernetics (SMC).

[31] Tan H.Z., Choi S., Lau F.W., Abnousi F. (2020). Methodology for maximizing information transmission of haptic devices: A survey. Proc. IEEE.

[32] Collins C.C. (1970). Tactile television-mechanical and electrical image projection. IEEE Trans. Man-Mach. Syst..

[33] Lin Y.N., Li Y.C., Ge S., Xu J.J., Li L.L., Xu S.Y. (2022). Three-Dimensional Encoding Approach for Wearable Tactile Communication Devices. Sensors.

[34] Van Erp J.B., Van Veen H. (2001). Vibro-tactile information presentation in automobiles. Proceedings of the Eurohaptics.

[35] Ricci F.S., Boldini A., Beheshti M., Rizzo J.R., Porfiri M. (2023). A virtual reality platform to simulate orientation and mobility training for the visually impaired. Virtual Real..

[36] Ricci F.S., Boldini A., Rizzo J.R., Porfiri M. (2023). A multiplayer virtual reality platform to evaluate electronic travel aid performance for persons with blindness and low vision. Nano-Bio -Info-Tech Sens. Wearable Syst..

[37] Chaudary B., Pohjolainen S., Aziz S., Arhippainen L., Pulli P. (2023). Teleguidance-based remote navigation assistance for visually impaired and blind people—Usability and user experience. Virtual Real..

[38] Slade P., Tambe A., Kochenderfer M.J. (2021). Multimodal sensing and intuitive steering assistance improve navigation and mobility for people with impaired vision. Sci. Robot..

[39] Bhatlawande S., Mahadevappa M., Mukherjee J., Biswas M., Das D., Gupta S. (2014). Design, development, and clinical evaluation of the electronic mobility cane for vision rehabilitation. IEEE Trans. Neural Syst. Rehabil. Eng..

[40] Khan M.A., Paul P., Rashid M., Hossain M., Ahad M.A.R. (2020). An AI-based visual aid with integrated reading assistant for the completely blind. IEEE Trans. Hum.-Mach. Syst..

[41] Kavanagh J.J., Menz H.B. (2008). Accelerometry: A technique for quantifying movement patterns during walking. Gait Posture.

[42] Reis D., Kupec J., Hong J., Daoudi A. (2023). Real-time flying object detection with YOLOv8. arXiv.

[43] Xie J., Yu R., Lee S., Lyu Y., Billah S.M., Carroll J.M. Helping helpers: Supporting volunteers in remote sighted assistance with augmented reality maps. Proceedings of the 2022 ACM Designing Interactive Systems Conference.

[44] Frisoli A., Leonardis D. (2024). Wearable haptics for virtual reality and beyond. Nat. Rev. Electr. Eng..

[45] Enayati N., De Momi E., Ferrigno G. (2016). Haptics in robot-assisted surgery: Challenges and benefits. IEEE Rev. Biomed. Eng..

